# Hepatic Artery Pseudoaneurysm Presenting as Gastrointestinal Hemorrhage

**DOI:** 10.7759/cureus.14190

**Published:** 2021-03-30

**Authors:** Pratishtha Singh, Nicolina Scibelli, Kiranpreet Gosal, Adam Bostick, Dylan C Morgan

**Affiliations:** 1 Internal Medicine, Grand Strand Regional Medical Center, Myrtle Beach, USA; 2 Internal Medicine, Grand Strand Medical Center, Myrtle Beach, USA; 3 Pulmonary and Critical Care Medicine, Grand Strand Medical Center, Myrtle Beach, USA

**Keywords:** hepatic artery pseudoaneurysm, gastrointestinal bleeding, hepatectomy, right hepatectomy

## Abstract

A hepatic artery pseudoaneurysm (HAP) is a rare complication of laparoscopic cholecystectomy. It can vary in its clinical presentation; however, given its severe nature, prompt assessment and management are crucial. We report a case of a 73-year-old male who underwent a laparoscopic cholecystectomy complicated by a right hepatic artery injury. This subsequently presented as a life-threatening case of upper gastrointestinal bleeding from HAP, with presumable hemobilia and septic shock from multiple liver abscesses. The diagnosis was made with computed tomography angiography (CTA) of the abdomen and pelvis followed by visceral angiography. The patient ultimately underwent a right hepatectomy for definitive treatment. The primary objective of this case is to highlight a less novel, though rare, case presentation and define a spectrum of treatment options available based on severity.

## Introduction

A hepatic artery pseudoaneurysm (HAP) is a rare complication of laparoscopic cholecystectomy. It can occur in relation to trauma, pancreatitis, percutaneous interventions, liver transplantation, and cholecystitis [1]. The true incidence is difficult to estimate, as many cases are asymptomatic or subclinical cases either thrombose spontaneously or are too small to detect on imaging [2]. The reported incidence after a laparoscopic procedure is estimated at 0.06% - 0.6% and involves the hepatic and cystic arteries [3], and approximately 50% of hepatic artery aneurysms are pseudoaneurysms [4]. This complication can vary in its clinical presentation; however, given its severe nature, it requires prompt assessment and management. If not identified, hemorrhage with rupture may occur, and mortality rates can be as high as 50% [5]. Common presenting symptoms include abdominal pain, jaundice, and gastrointestinal bleed while many patients may be asymptomatic. HAP with hemobilia can be diagnosed with either endoscopic or radiological imaging. Treatment typically involves embolization, although, in the presence of complications, surgery may be indicated.

## Case presentation

A 73-year-old male with a medical history of hypertension, coronary artery disease, and chronic alcohol use presented to the emergency department (ED) with fever, melena, and intermittent episodes of confusion. The patient was admitted to the hospital seven weeks prior to this admission for an elective laparoscopic cholecystectomy. His course was complicated by a right hepatic artery injury treated with ligation and clipping, with subsequent development of multiple hepatic abscesses, Escherichia coli, Bacteroides fragilis, Enterococcus faecium bacteremia, and a bile leak. The bile leak required treatment with endoscopic retrograde pancreatography (ERCP) with sphincterotomy and a common bile duct (CBD) stent placement. The patient was discharged to rehab with a Jackson Pratt (JP) drain (Cardinal Health, Dublin, Ohio) in place. He was continued on daptomycin and ertapenem for four weeks for hepatic abscesses per the infectious disease specialist. The patient returned to the hospital for recurrent fevers and melena. History was limited due to acute delirium but right upper quadrant abdominal pain was noted. He denied bright red blood per rectum, hematemesis, nausea, vomiting, or nonsteroidal anti-inflammatory drug (NSAID) use. His JP drain had a recorded output of approximately 30-40 cc/day.

The patient was febrile with a temperature of 101.7 Fahrenheit within a few hours of admission. Other vital signs were significant for sinus tachycardia, with 110 beats per minute, and tachypnea, with 22 breaths per minute. Blood pressure and oxygen saturation were at 154/65 and 100% on room air, respectively. The physical exam was notable for confusion, abdominal tenderness to palpation that was worse in the right upper quadrant, and the JP drain in the right upper quadrant. Laboratory findings on admission were significant for leukocytosis, with a white blood cell (WBC) count of up to 13,200/mm^3^. His hemoglobin and hematocrit were noted to be at 6.2 gm/dL and 19%, respectively, which was decreased from the hemoglobin of 10.3 gm/dL on the day of his last discharge. Platelets on admission were within normal limits at 213,000/mm^3^. His comprehensive metabolic panel from admission revealed transaminitis, with an elevated aspartate aminotransferase of 149 U/L, alanine aminotransferase of 134 U/L, and alkaline phosphatase at 51 0U/L. There was no significant elevation of total bilirubin. The patient's prothrombin time was elevated to 16.1 seconds, and the international normalized ratio INR was 1.41 on admission.

On day one, the patient became hemodynamically unstable, with worsening mentation, tachycardia, and tachypnea, requiring a transfer to the intensive care unit. Surgery and gastrointestinal medicine were consulted for evaluation. The patient underwent imaging with computed tomography (CT) of the abdomen and pelvis, which revealed persistent postsurgical changes of cholecystectomy and an unchanged common bile duct stent. A right-sided abdominal drain was noted to be in the right upper quadrant. Multilobulated, predominantly hypoattenuating masses were noted throughout the liver, with persistent hyperdensity, more centrally suggestive of combined multifocal hepatic abscess and central hemorrhage, also reported. He was transfused with 2 units of packed red blood cells (RBCs) and resuscitated with intravenous (IV) lactated ringer fluids. Blood cultures were obtained, and the patient was started on broad-spectrum antibiotics.

**Figure 1:**
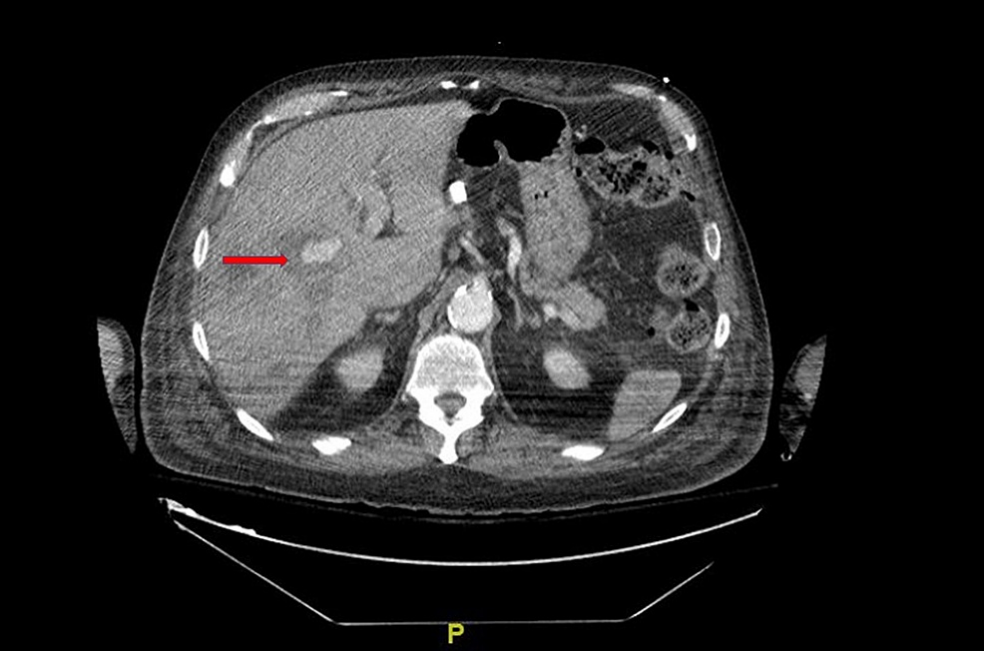
Axial view hepatic artery pseudoaneurysm seen on CTA abdomen Red arrow pointing to the hepatic artery pseudoaneurysm as seen on an axial view on CTA abdomen. computed tomography angiography (CTA)

He was taken for an esophagoduodenoscopy (EGD) to assess for causes of upper gastrointestinal bleeding (UGIB). This did not show any evidence of active bleeding or hemobilia at the previous sphincterotomy site; however, the patient had significant melena noted at the end of the exam and was emergently taken for further evaluation with computed tomographic angiography (CTA). CTA revealed transient hyperdensity within the right lobe of the liver, adjacent to the right portal vein, suspicious for a hepatic artery injury/pseudoaneurysm as seen in Figure 1.

**Figure 2:**
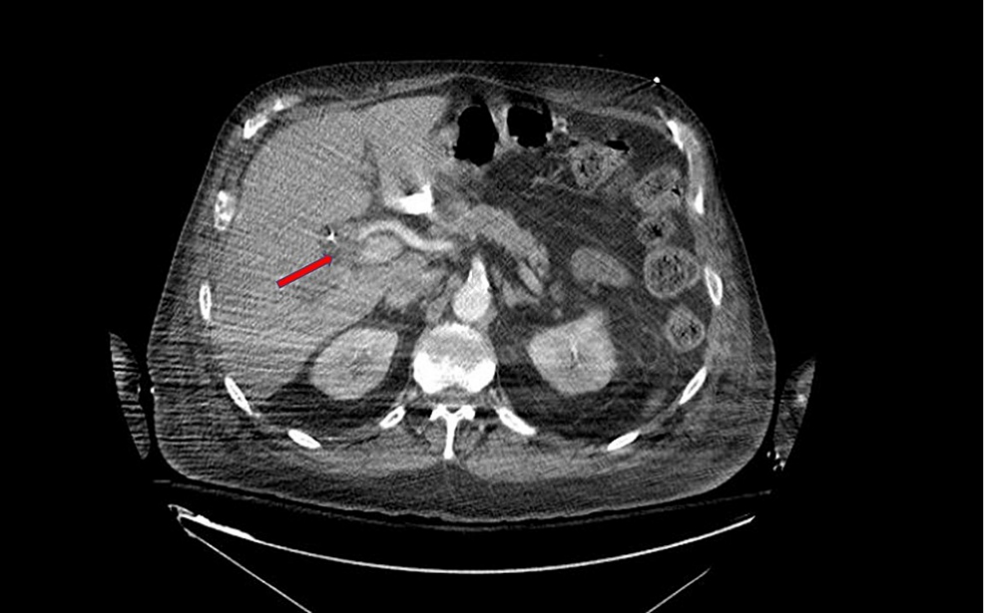
Axial view of clipped right hepatic artery seen on CTA abdomen This figure demonstrates the common hepatic artery branching into the right and left hepatic arteries. A red arrow is pointing toward the right hepatic artery, which was ligated and clipped during the patient's initial surgery. computed tomography angiography (CTA)

Figure 2 shows CTA findings of the clipped right hepatic artery from the patient's initial surgery.

**Figure 3:**
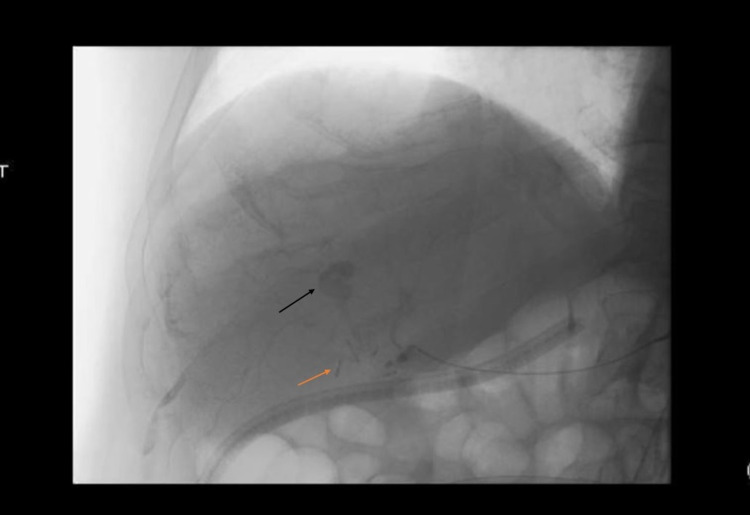
Common hepatic angiogram revealing hepatic artery pseudoaneurysm Images obtained during visceral angiography with the orange arrow indicating the prior clipping of the right hepatic artery and the black arrow indicating the pseudoaneurysm.

**Figure 4:**
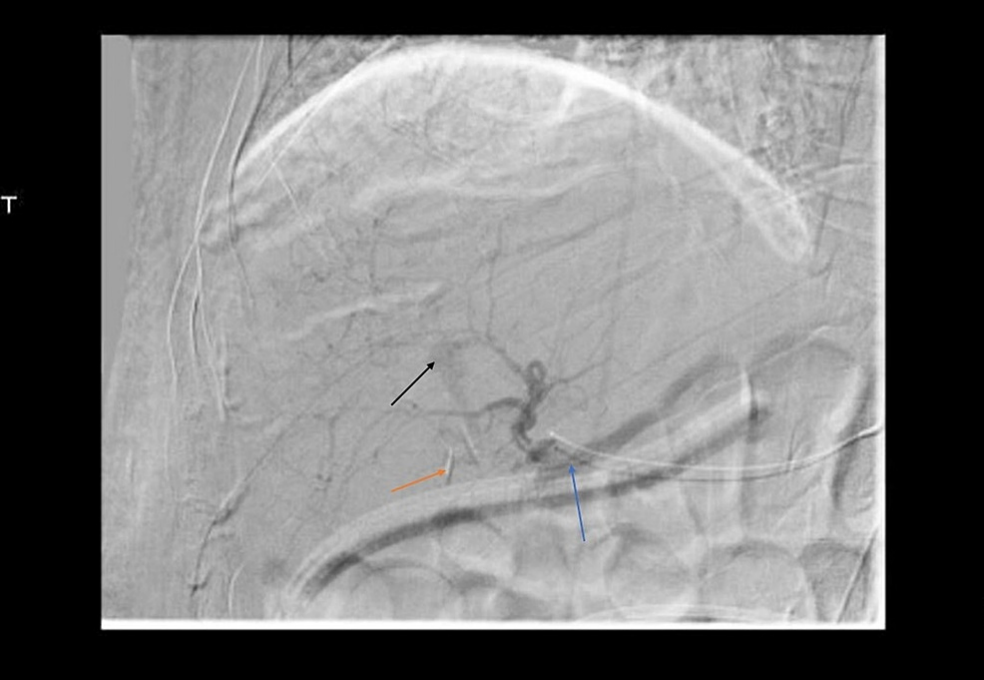
Digital subtraction angiography of the common hepatic artery This image shows digital subtraction angiography of the common hepatic artery with the black arrow pointing to the hepatic artery pseudoaneurysm, the orange arrow pointing to prior ligation and clips placed on the right hepatic artery, and the blue arrow pointing to the common hepatic artery.

He was then taken for visceral angiography by interventional radiography (IR), which confirmed a previous iatrogenic hepatic artery ligation and showed an arterial enhancing structure within the central aspect of the liver consistent with a hepatic artery pseudoaneurysm, as seen in Figure 3 and Figure 4.

The patient’s pseudoaneurysm was not amenable to catheter-directed embolization, as there was no direct feeding arterial structure identified. He was subsequently taken to the operating room for a right hepatectomy. Post right hepatectomy, the patient remained tenuous and began to decline quickly after developing acute oliguric renal failure, which required continuous renal replacement therapy (CRRT) and resistant hypoglycemia. Additionally, worsening shock required pressure and, ultimately, disseminated intravascular coagulopathy (DIC). Due to the patient’s acute decompensation and prolonged hospital course, his wife elected to move toward comfort care and withdrawal of aggressive measures.

## Discussion

Visceral artery aneurysms can be true aneurysms or pseudoaneurysms. True aneurysms are outpouchings containing all three layers of blood vessel walls (intima, media, and adventitia). Pseudoaneurysms are outpouchings that do not contain at least one of these layers due to some form of wall injury. Some commonly involved arteries for pseudoaneurysm presentation include the splenic artery (most common), gastroduodenal artery, pancreatoduodenal artery, superior mesenteric, left gastric, hepatic, and small intrapancreatic arteries [6].

HAP is a rare complication of laparoscopic cholecystectomy that can lead to life-threatening bleeding and infection. The incidence of vascular injury during cholecystectomy is largely unknown, as most studies look at a combined biliary and vascular injury, but an autopsy study found the rate of right hepatic artery injury after open cholecystectomy to be 7% [7-8]. Predisposing factors for pseudoaneurysm formation after surgery include digestion of the hepatic arterial wall by infectious bile leak, arterial irritation from a localized abscess, and mechanical injury of the artery during surgery [9]. Our patient was at high risk for pseudoaneurysm formation, as he had a known hepatic artery ligation injury during a laparoscopic cholecystectomy, and his post-surgical course was complicated by bile leak, abscess formation, and bacteremia. Additionally, our patient was presumed to have an arterio-biliary fistula complicating his pseudoaneurysm due to the heavy amount of melena and radiographic findings, despite no hemobilia seen on EGD. This likely indicates arterial wall damage from a bile leak was also contributing to pseudoaneurysm formation.

HAPs can be found incidentally but often present hemobilia (90%), abdominal pain (70%), and jaundice (60%). Less than 40% present with the classic Quincke's Triad (jaundice, biliary colic, and gastrointestinal bleeding) [2]. Our patient presented with upper gastrointestinal bleeding and abdominal pain, but no jaundice. Management of acute gastrointestinal bleeding involves identifying the source of the bleed, for which endoscopy is the preferred method but CTA is also used [10]. Our patient first underwent endoscopy, which showed no active bleeding; there was also no mention of dried blood. Given the patient's continued melena, a CTA of the abdomen and pelvis was performed, which provided strong evidence for HAP but was not definitive per the radiology report. Finally, visceral angiography confirmed the diagnosis of HAP and helped guide the path towards treatment.

HAP is considered an acute emergency requiring immediate intervention. The risk of pseudoaneurysm rupture is 21%-80%, with an associated mortality of up to 50% [2,5]. Minimally invasive techniques are both successful and preferred to prevent complications. The first-line treatment for HAP is urgent selective hepatic arterial angiography and embolization. The benefits of angiography and embolization include speed, targeted therapy, minimal invasiveness, and potential for repetition, if necessary. The risks include embolization into other arteries and hepatic ischemia. A second treatment option is ultrasound-guided percutaneous thrombin injection, which can decrease the risk of hepatic infarction [11]. This option has been described in limited case reports and could be considered on a case-by-case basis [2,12-13].

Surgery is typically not the initial recommendation for treatment, as it can lead to adhesions and altered anatomy. If surgery is required, such as in the event of repeated failed embolization or no identifiable direct arterial feeding structures, it is typically performed by the ligation of feeding vessels and excision of the HAP [14]. Our patient’s pseudoaneurysm had no direct arterial feeding structure so was not a candidate for endovascular coil embolization. Our patient's pseudoaneurysm was also complicated by multiple hepatic abscesses, a phenomenon that has rarely been described in the literature but has been described with amoebic liver abscess [15-17]. Our patient's blood cultures grew various gram-negative organisms during his stay, including Escherichia coli, Bacteroides fragilis, and Enterococcus faecium, which were treated with daptomycin and ertapenem, and presumably were the organisms causing his hepatic abscesses. Percutaneous thrombin embolization was considered after a liver ultrasound identified the likely pseudoaneurysm, but open right hepatectomy was ultimately pursued, as it allowed for simultaneous excision of the HAP and hepatic abscesses.

## Conclusions

A HAP can be a complication of cholecystectomy in which patients can be asymptomatic or present with abdominal pain, jaundice, and gastrointestinal bleeding. Prompt diagnosis should be made with endoscopy to localize bleeding and to rule out other causes of UGIB. If not definitive or with evidence of hemobilia, we can obtain CTA to assist with diagnosis. Treatment options include embolization, a percutaneous thrombin injection, or surgery. We present a case of HAP occurring as a complication after elective cholecystectomy presenting as a life-threatening bleed. Patients with HAP are at high risk of decline due to bleeding and infection, making early recognition and definitive treatment extremely important.
